# Supplementary material for: Expression of FoxP2 in the basal ganglia regulates vocal motor sequences in the adult songbird
Source: Nat Commun. 2021 May 11;12:2617. doi: 10.1038/s41467-021-22918-2 (PMC8113549; doi:10.1038/s41467-021-22918-2)
Supplement: Supplementary file 1 — Supplementary Information [file 41467_2021_22918_MOESM1_ESM.pdf]

Expression of FoxP2 in the Basal Ganglia Regulates Vocal Motor Sequences in the Adult Songbird

Lei Xiao[1], Devin P. Merullo[1], Therese M. I. Koch[1], Mou Cao[2], Marissa Co[1], Ashwinikumar Kulkarni[1], Genevieve Konopka[1], and Todd F. Roberts[1#]
[1]Department of Neuroscience UT Southwestern Medical Center, Dallas, TX, USA.
[2]Department of Pediatrics, UT Southwestern Medical Center, Dallas, TX, USA.
[#]Correspondence should be addressed to TFR (Todd.Roberts@utsouthwestern.edu)

SUPPLEMENTAL INFORMATION:

Supplementary Figures 1 to 12

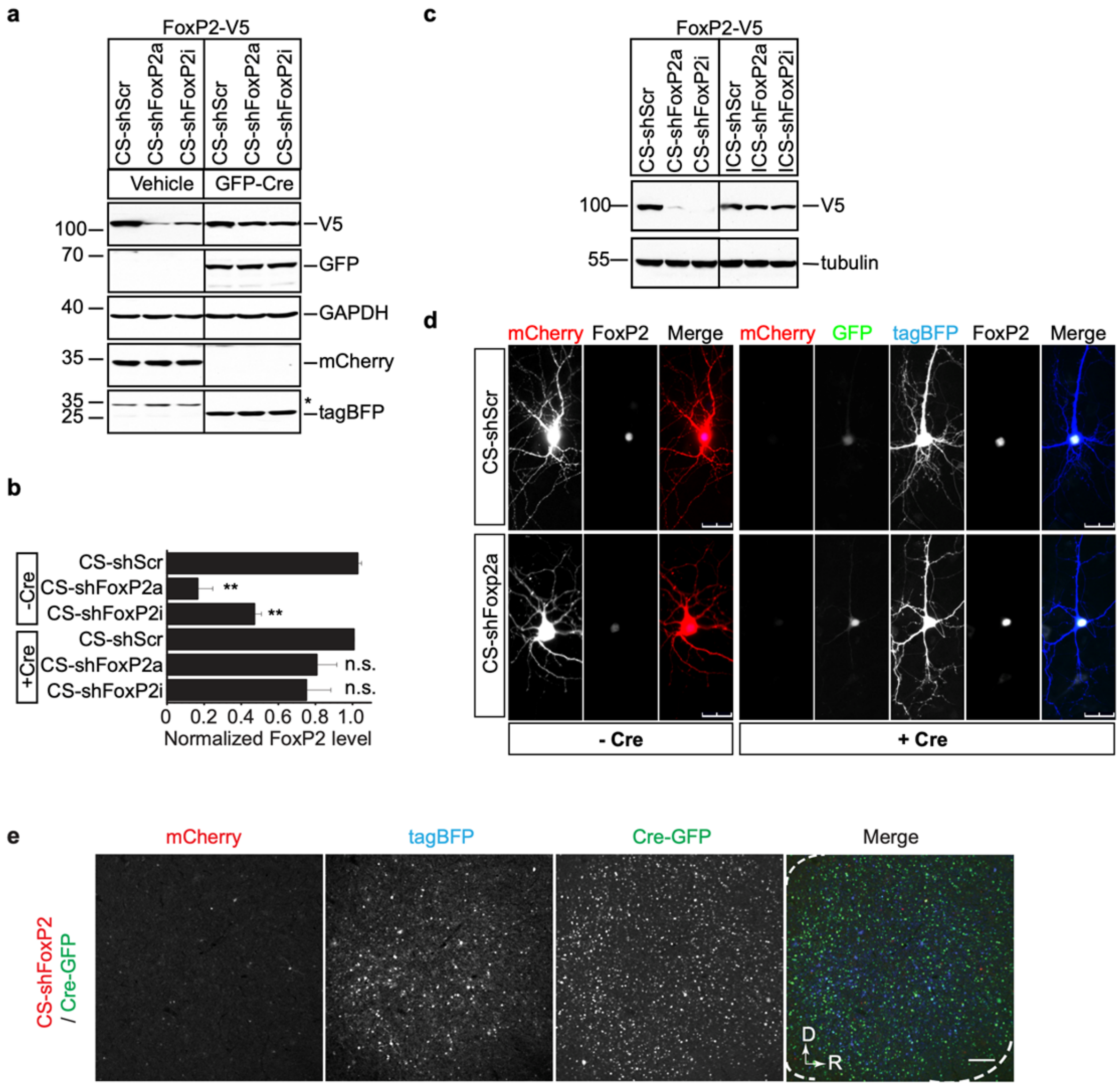

Supplementary Figure 1. Validation of CS constructs *in vitro* and *in vivo*.

(a) Validation of CS constructs *in vitro* with western blotting. Two independent small hairpin RNAs against the zebra finch FoxP2 gene (CS-shFoxP2a & i) and scrambled hairpin (CS-shScr), together with V5-tagged zebra finch FoxP2, were co-transfected into HEK293T stable cell lines expressing either vehicle or GFP-Cre as indicated. At 72 h after transfection, lysates of cells were subjected to immunoblotting with V5 (to detect FoxP2), GFP (to detect Cre-GFP), GAPDH, RFP (to detect mCherry) and tagBFP antibody.
(b) The expression levels of FoxP2 were quantified from three independent experiments as shown in (a). CS-shFoxP2a and CS-shFoxP2i constructs resulted in equivalent downregulation of FoxP2 protein levels ($p<0.0001$ and $p=0.0004$, ANOVA), which were rescued in the presence of Cre recombinase ($p=0.08$ and $p=0.93$, ANOVA).
(c) Validation of ICS constructs *in vitro* with western blotting. ICS constructs were generated and purified from an *in vitro* cre recombination assay. Both CS and ICS constructs, together with V5-tagged zebra finch FoxP2, were co-transfected into HEK293T cell as indicated. At 72 h after transfection, lysates of cells were subjected to immunoblotting with V5 (to detect FoxP2) and tubulin antibody. In contrast to both CS-shFoxP2 constructs which were sufficient to downregulate FoxP2 protein level, neither ICS-shFoxP2 construct resulted in any change in FoxP2 expression levels.
(d) Validation of CS constructs in primary culture. CS-shScr or CS-shFoxP2, together with V5-tagged zebra finch FoxP2, were co-transfected into mouse cortical primary culture with or without Cre-GFP (+Cre & -Cre respectively). For both the CS-shScr and CS-shFoxP2 constructs, the expression of mCherry was maintained in the absence of Cre, whereas the expression of mCherry was turned off and BFP was turned on in the presence of Cre. Scale bar, 30 μm.
(e) Representative parasagittal section shows the expression patterns of mCherry and tagBFP in Area X of an adult bird injected with CS-shFoxP2 and Cre-GFP constructs. Dashed lines outline the border of Area X. D, dorsal; R, rostral. Scale bar, 100 μm.

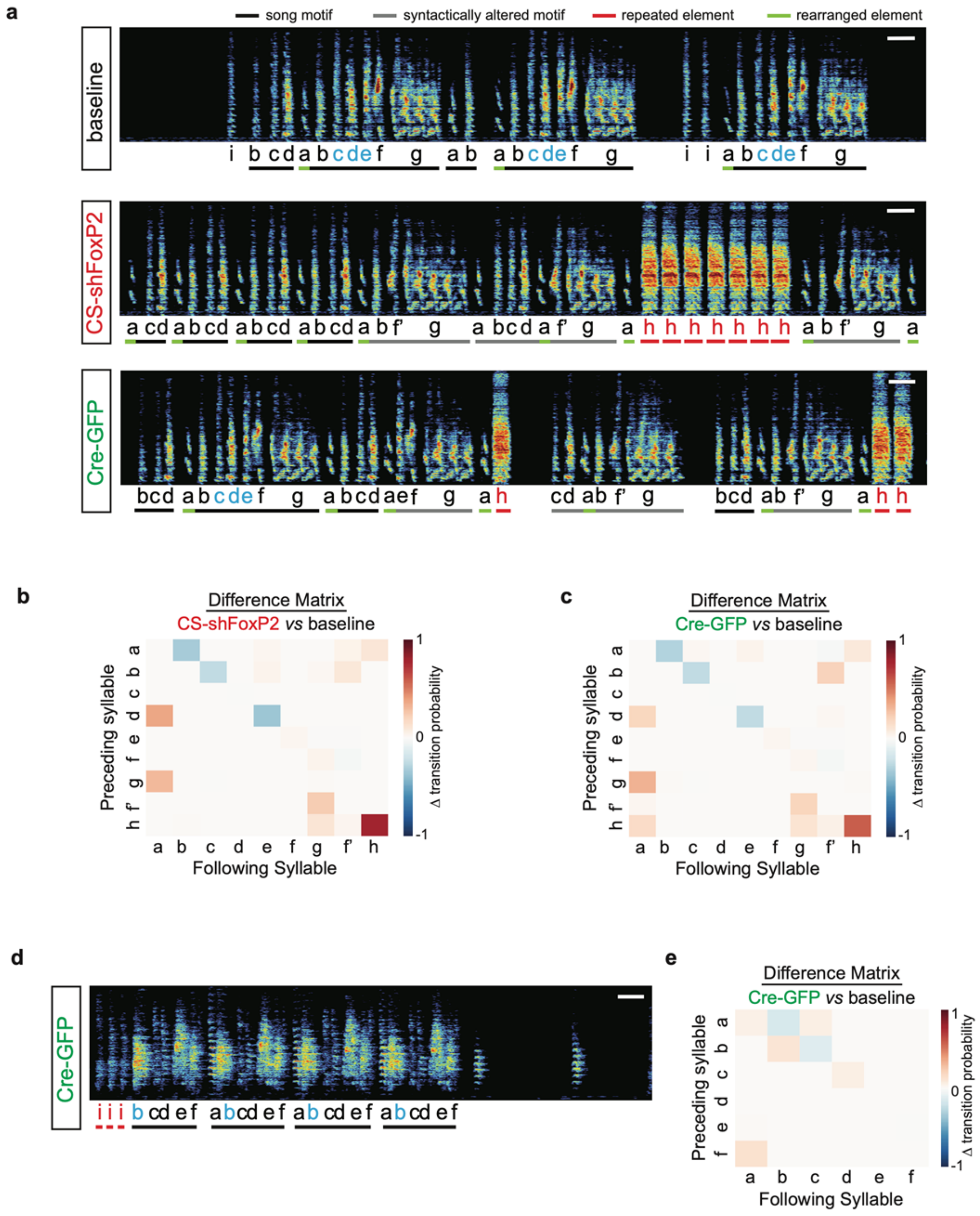

Supplementary Figure 2. Changes in song following knockdown and of FoxP2 in Area X.

(a) Spectrograms of song recorded at baseline, 2 months after injection of CS-shFoxP2, and 2 months after injection of Cre-GFP in Area X of an adult bird. A *de novo* syllable 'h' emerged one-month following injection of CS-shFoxP2, and the high number of repetitions of this syllable was maintained for up to four months (data not shown) post injection of CS-shFoxP2. In a subset of motifs, a portion of motif (syllables 'cde', blue) was omitted following injection of CS-shFoxP2. In another subset of motifs, the syllable 'e' was replaced with syllable 'a'(green). The de novo syllable 'h' was retained at the end of the motif, whereas the number of repetitions in each song bout was significantly decreased 2 months following injection of Cre-GFP. All other changes in song caused by FoxP2 knockdown were not rescued 2 months following injection of Cre-GFP. Each letter indicates an individual syllable. Syllable 'f'' is considered as a variant of syllable 'f'. Scale bar, 200ms.

(b)&(c) difference transition matrices for the bird shown in (a). Subtracting the syllable transition matrix at 2 months following CS-shFoxP2 injection or at 2 months following Cre-GFP injection from the matrix at baseline reveals changes in the song sequence during reversable knockdown of FoxP2.

(d) Spectrograms of one bout of song recorded two months after bilateral injection of Cre-GFP construct in Area X of an adult bird which was injected with CS-shFoxP2 2 months prior (previous spectrograms were shown in Figure 2d). The number of repetitions of

introductory elements in each song bout was restored to baseline level, and a previously omitted syllable was recovered 2 months following injection of Cre-GFP.

(e) Difference transition matrices for the bird shown in Figure 2d. Subtracting the song transition matrix at 2 months following Cre-GFP injection (d) from the matrix at baseline (Figure 2d) reveals little changes in the song sequence of the bird following reversable knockdown of FoxP2.

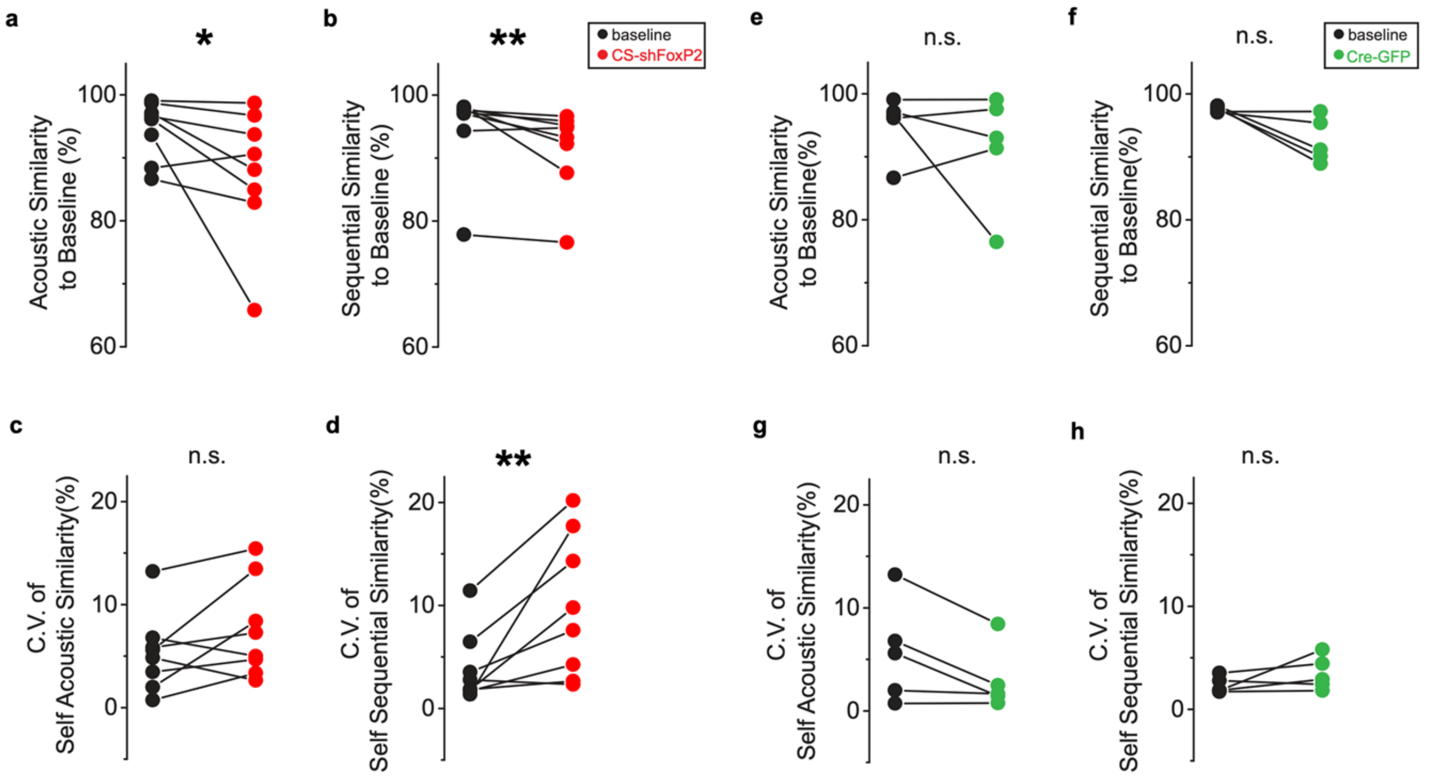

Supplementary Figure 3. Song similarity during reversable knockdown of FoxP2 in Area X.
(a) Acoustic similarity scores at baseline (94.53 ±1.65%) and two months post injection of CS-shFoxP2 (87.68.34 ±3.68%). Knockdown of FoxP2 in Area X resulted in a significant decrease in acoustic similarity to baseline ($p = 0.039$, $n = 8$, Wilcoxon signed-rank test).
(b) Sequential similarity scores at baseline (94.72 ±2.45%) and two months post injection of CS-shFoxP2 (91.53±2.36%). Knockdown of FoxP2 in Area X resulted in a significant decrease in sequential similarity to baseline ($p = 0.016$, $n = 8$, Wilcoxon signed-rank test).
(c) Variability in acoustic self-similarity at baseline (CV = 5.3 ±1.34%) and two months post injection of CS-shFoxP2 (CV = 7.53 ±1.66%). Knockdown of FoxP2 in Area X did not change the CV of acoustic self-similarity ($p = 0.15$, $n = 8$, Wilcoxon signed-rank test).
(d) Variability in sequential self-similarity at baseline (CV = 3.87 ±1.23%) and two months post injection of CS-shFoxP2 (CV = 9.85 ±2.44%). Knockdown of FoxP2 in Area X resulted in a significant increase in the CV of sequential self-similarity ($p = 0.016$, $n = 8$, Wilcoxon signed-rank test).
(e) Acoustic similarity scores at baseline (95.1 ±2.17%) and two months post injection of Cre-GFP in CS-shFoxP2+ birds (91.5±4.02%). Acoustic similarity scores following reversal of FoxP2 expression in Area X were not significantly different from scores at baseline ($p > 0.99$, $n = 5$, Wilcoxon signed-rank test).
(f) Sequential similarity scores at baseline (97.57 ±0.2%) and two months post injection of Cre-GFP in CS-shFoxP2+ birds (92.55±1.6%). Sequential similarity scores following reversal of FoxP2 expression in Area X were not significantly different from scores at baseline ($p = 0.13$, $n = 5$, Wilcoxon signed-rank test).
(g) Variability in acoustic self-similarity at baseline (CV= 5.66 ±2.19%) and two months post injection of Cre-GFP in CS-shFoxP2+ birds (CV= 2.95 ±1.4%). The CV of acoustic self-similarity following reversal of FoxP2 expression in Area X was not significantly different from the CV at baseline ($p = 0.13$, $n = 5$, Wilcoxon signed-rank test).
(h) Variability in sequential self-similarity at baseline (CV= 2.34 ±0.35%) and two months post injection of Cre-GFP in CS-shFoxP2+ birds (CV= 3.47 ±0.73%). The CV of self- sequential similarity following reversal of FoxP2 expression in Area X was not significantly different from the CV at baseline ($p = 0.19$, $n = 5$, Wilcoxon signed-rank test).

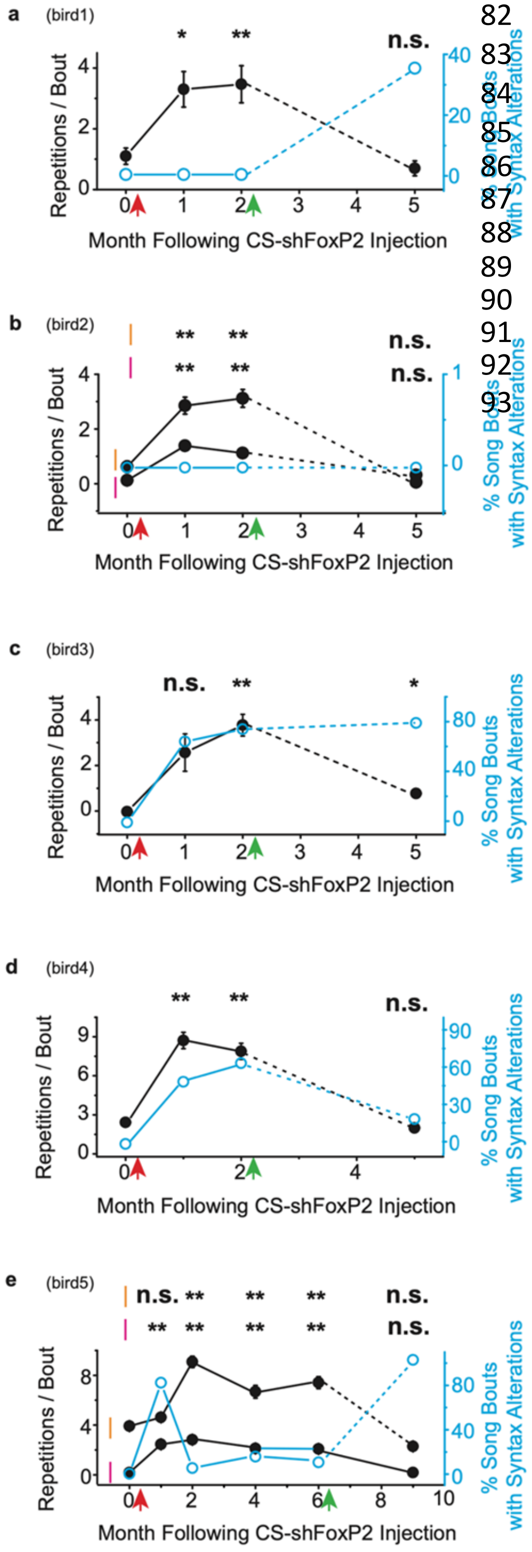

Supplementary Figure 4. Reversal of FoxP2 knockdown in adult zebra finches rescues syllable repetitions but not changes in song syntax.

Changes in the number of syllable repeats (black) and % of song bouts exhibiting syntax alterations (blue) for five CS-shFoxP2+ birds (a-e, each panel represents an individual bird) who were injected with Cre-GFP (green arrow) 2-6 months after the initial knockdown of FoxP2 (red arrow). We identified two individual syllables were uncharacteristically repeated following CS-shFoxP2 injections in birds 2 and 5 (panels b & e). Each syllable is plotted separately (indicated by orange and purple lines).

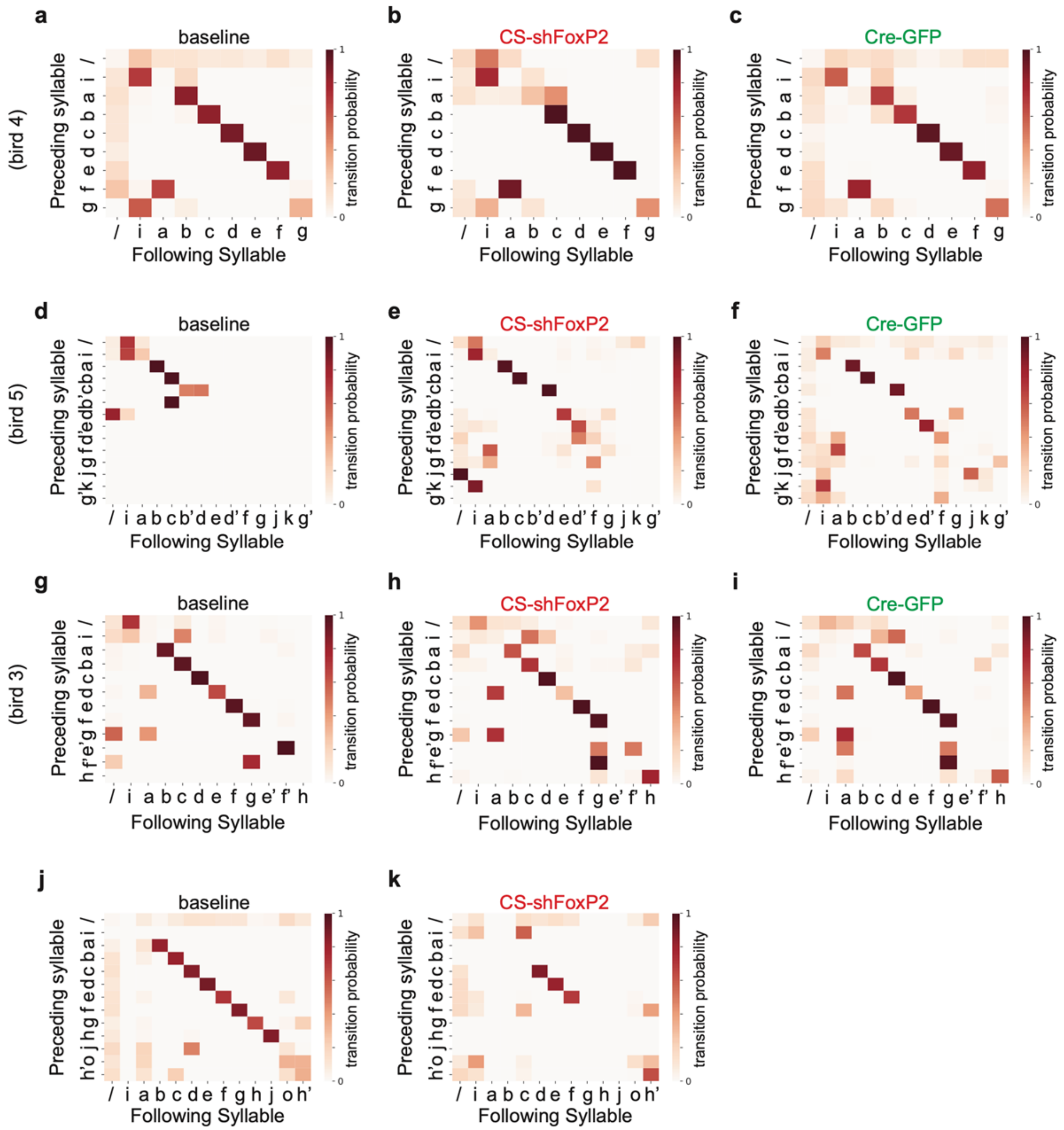

Supplementary Figure 5. Syllable transition matrices

(a)-(c) Syllable transition matrices for the bird shown in Figure 2d (bird 4 in Figure S4d) at baseline(a), 2 months following CS-shFoxP2 injection (b), and 2 months following Cre-GFP injection (c). Color indicates the probability of a transition from the preceding syllable along the y axis to the following syllable along the x axis. "/", reflects a silent gap of over 100 ms, and is considered the beginning or end of a motif.

(d)-(f) Syllable transition matrices for the bird shown in Figure 3e (bird 5 in Figure S4e) at baseline (d), 4 months following CS-shFoxP2 injection(e), and 3 months following Cre-GFP injection (f).

(g)-(i) Syllable transition matrices for the bird shown in Figure S2a (bird 3 in Figure S4c) at baseline (g), 2 months following CS-shFoxP2 injection (h), and 2 months following Cre-GFP injection (i).

(j)&(k) Syllable transition matrices for the bird shown in Figure 2e at baseline (j) and 2 months following CS-shFoxP2 injection (k).

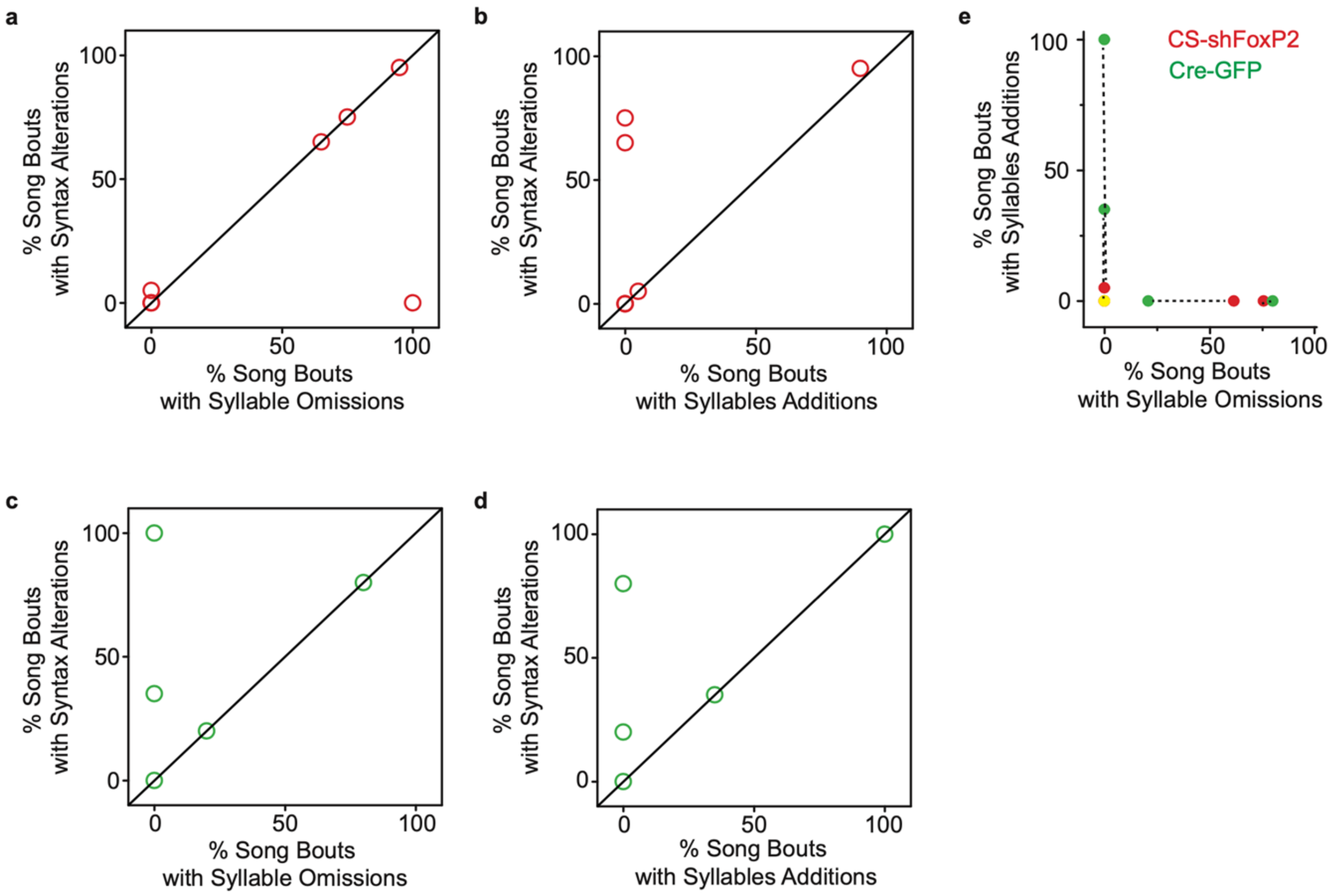

Supplementary Figure 6. Relationship between syllable addition or omission and syntax alteration in CS-shFoxP2+ birds before and after reversal of FoxP2 knockdown.

(a) The % of syllable omission and syntax alteration 2 months after injection of CS-shFoxP2 (n=8 birds, each circle represents an individual bird).

(b) The % of syllable addition and syntax alteration 2 months after injection of CS-shFoxP2 (n=8 birds, each circle represents an individual bird).

(c) The % of syllable omission and syntax alteration 2 months after injection of Cre-GFP in CS-shFoxP2+ birds (n=5, each circle represents an individual bird).

(d) The % of syllable addition and syntax alteration 2 months after injection of Cre-GFP (n=5, each circle represents an individual bird).

(e) Changes in the % of syllable omission and addition in CS-ShFoxP2+ birds (n=5) before (red circle, CS-shFoxP2+) and 2 months after injection of Cre-GFP (green circle, Cre-GFP+). Dashed lines connect the same bird before and after reversal of FoxP2 knockdown. The yellow circle represents birds that didn't display syllable addition or omission either before or after reversal of FoxP2 knockdown.

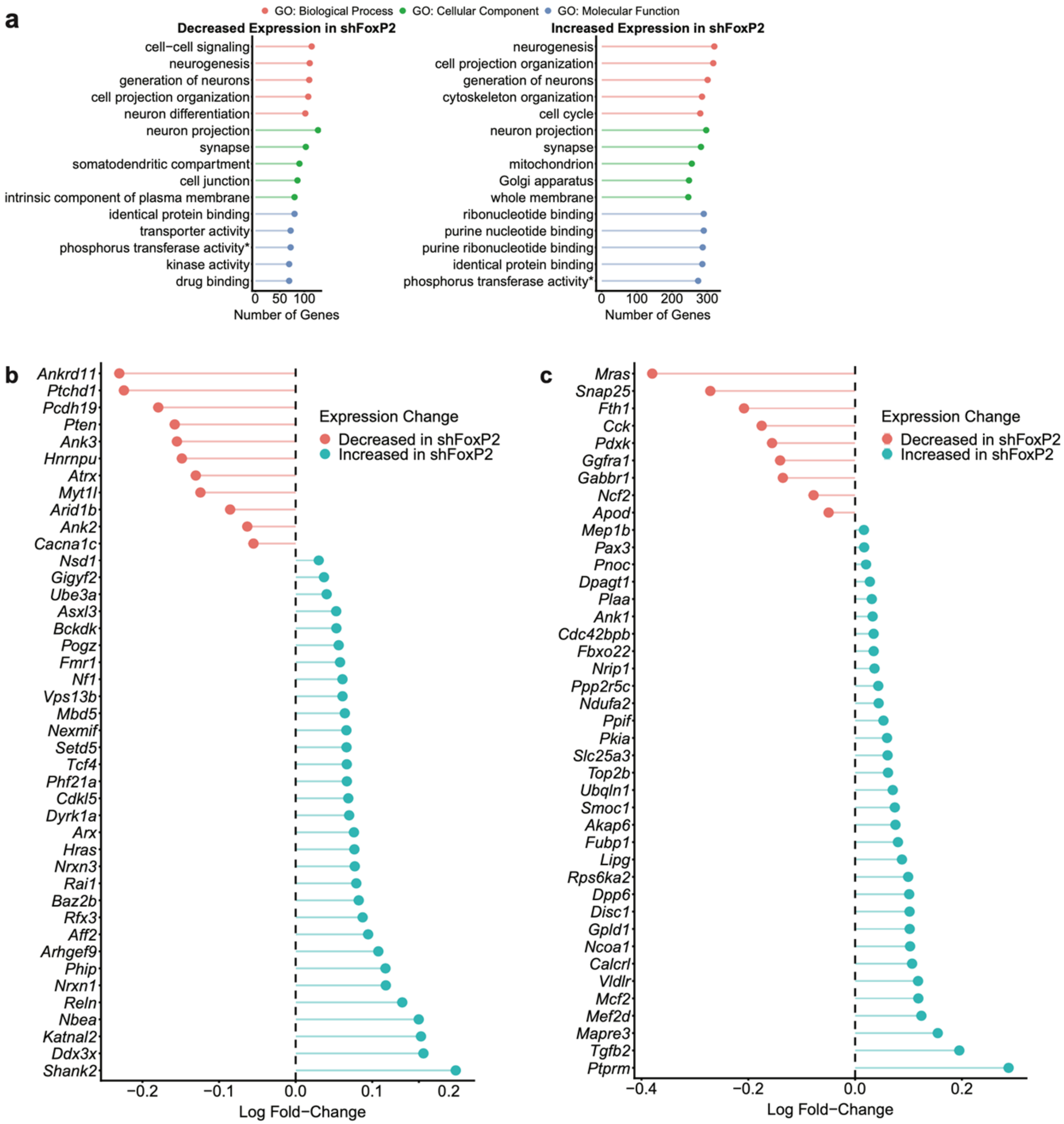

Supplementary Figure 7. Gene expression differences in FoxP2+ cells

(a) The top five most significant categories of genes affected in FoxP2+ cells of CS-shFoxP2+ birds for each of the three main gene ontology classifications: Biological Process, Cellular Component, and Molecular Function.

(b) Log fold-changes in expression of genes in FoxP2+ cells of CS-shFoxP2+ birds that are highly scored autism-related genes by the SFARI Gene database.

(c) Log fold-changes in expression of genes in CS-shFoxP2+ birds identified previously as direct transcriptional targets of FOXP2.

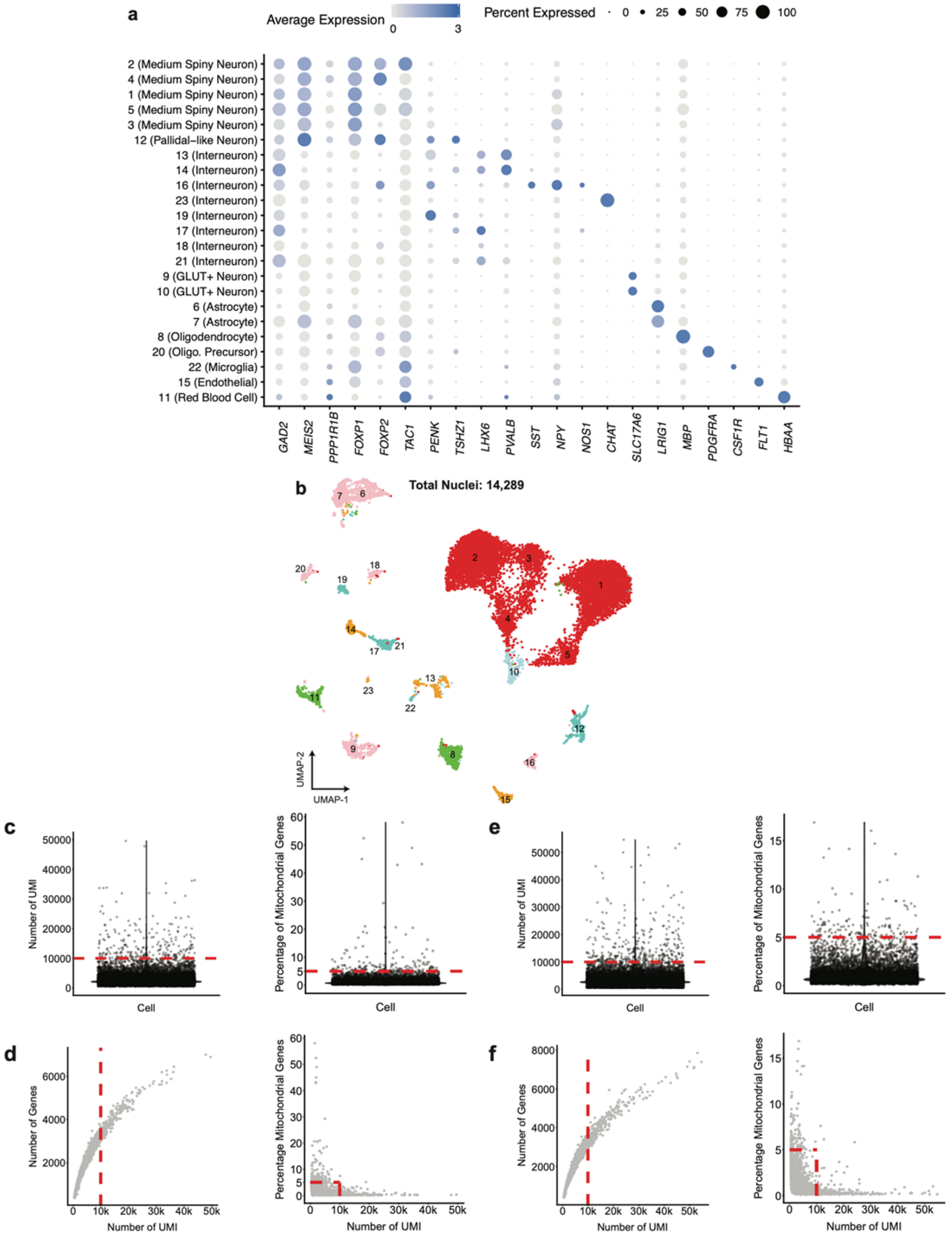

Supplementary Figure 8. Details of single-cell transcriptomics.

(a) A heatmap of normalized expression for genes used to assign identities to cell types for a combined analysis of the CS-shScr+ and CS-shFoxP2 datasets (corresponding to Figure 4a). Expression was normalized globally across all genes, but a different scale is shown for each gene based on the highest normalized value. For marker gene expression, the size of the dot indicates the percent of nuclei within a cluster expressing a given gene, and the color of the dot indicates the average normalized expression level.

(b) A UMAP projection of nuclei from Area X of an independent analysis of CS-shScr+ birds (not merged, corresponding to Figure 4c). Clusters are numbered in ascending order by decreasing size (1-largest; 23-smallest).

(c) For CS-shScr+, density plot of the number of UMIs per cell (left) and the percentage of mitochondrial genes in each cell (right). The analysis only included cells with UMI < 10,000 and < 5% mitochondrial genes (indicated by the red dashed line).

(d) For CS-shScr+, a scatterplot of the number of UMIs per nucleus and the number of genes (left) or percentage of mitochondrial genes (right). Each dot is a cell. The cells within the red dashed box, corresponding to the filters in (c), were the cells analyzed.

(e) For CS-shFoxP2+, density plot of the number of UMIs per cell (left) and the percentage of mitochondrial genes in each cell (right). The analysis only included cells with UMI < 10,000 and < 5% mitochondrial genes (indicated by the red dashed line).

(f) For CS-shFoxP2+, a scatterplot of the number of UMIs per nucleus and the number of genes (left) or percentage of mitochondrial genes (right). Each dot is a cell. The cells within the red dashed box, corresponding to the filters in (e), were the cells analyzed.

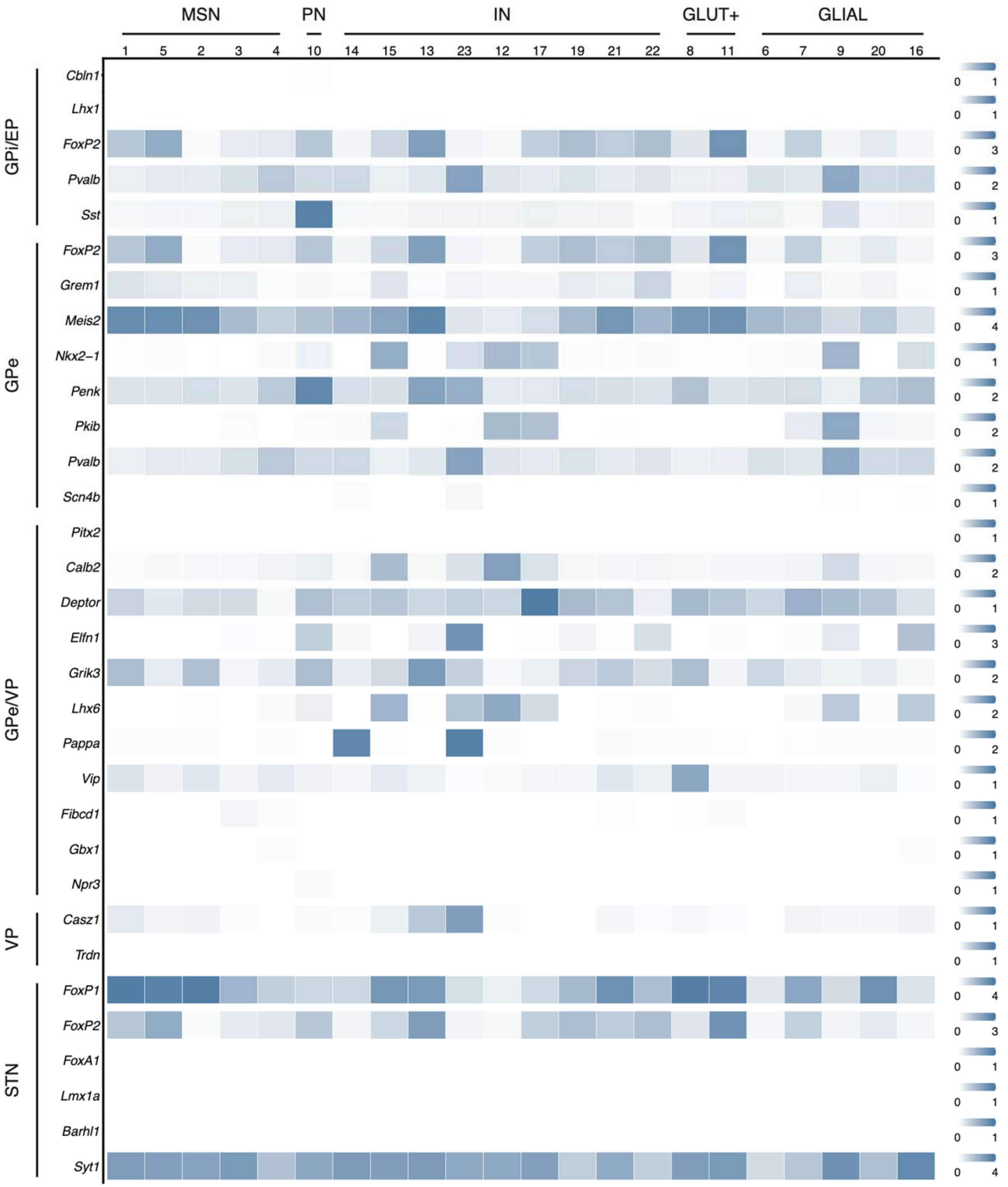

Supplementary Figure 9. Supplemental gene markers of basal ganglia regions.

As in Figure 4c, expression was normalized globally across all genes, but a different scale is shown for each gene based on the highest normalized value. Gene markers were selected from published studies (GPi/EP [1,2]; GPe [1,3]; GPe/VP [1]; VP [1]; STN [1,4]).

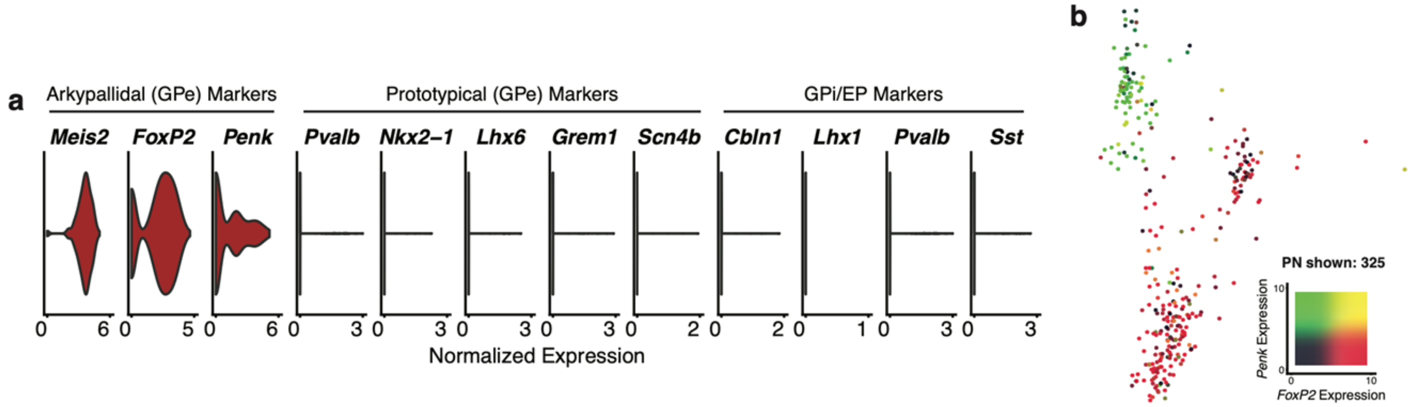

Supplementary Figure 10. Gene markers of arkypallidal cells

(a) A violin plot showing normalized expression in pallidal-like cells of gene markers for arkypallidal GPe cells, prototypical GPe cells, and the GPi/EP.

(b) UMAP projection of pallidal-like cells, modified from Figure S8, with each nucleus colored according to the normalized expression of FoxP2 and Penk. Expression value colors are relative to each gene (0-lowest value for that gene; 10-highest value for that gene). Unlike arkypallidal cells, the pallidal-like cells in Area X do not co-express FoxP2 and Penk but instead form separate populations.

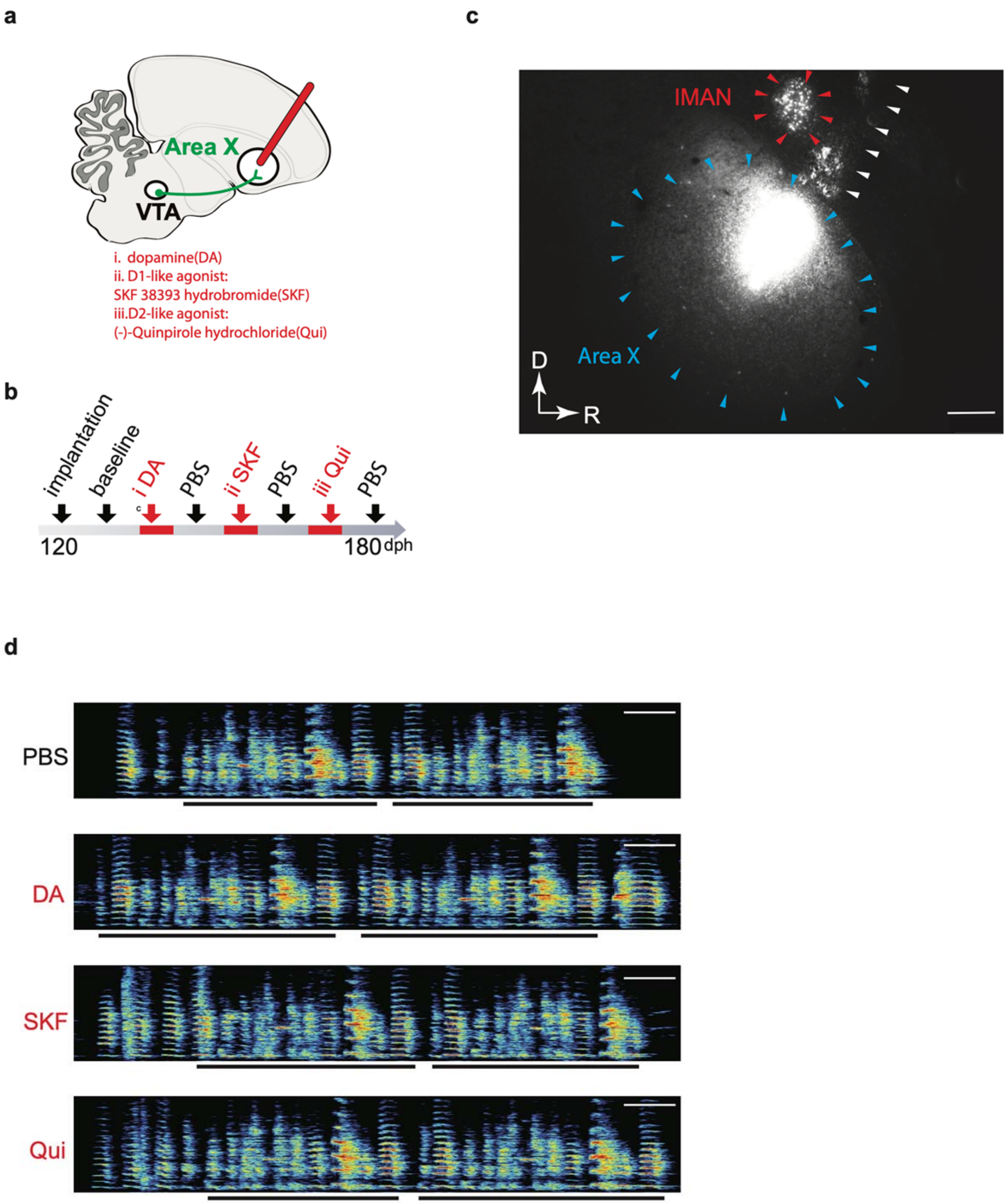

Supplementary Figure 11. Lack of changes in syllable repetitions with pharmacological manipulation of dopamine or dopamine receptors.

(a-b) Schematic of experimental design. Dopamine (DA, i), D1-like agonist SKF 38393 hydrobromide (SKF, ii.) and D2-like agonist (-)-Quinpirole hydrochloride (Qui, iii.) were infused bilaterally into Area X of behaving adult birds through a microdialysis system. Each chemical was delivered individually for 4-8 days, one week apart from each other.

(c) Parasagittal section showing the implantation track of a microdialysis probe in Area X, and the anterior to lateral magnocellular nucleus of the anterior nidopallium (lMAN). A fluorescent retrograde tracer Fast blue was delivered to Area X via the microdialysis probe at the end of each experiment to visualize the field of pharmacological manipulation. The fluorescent signals indicate the center of Fast blue infusion in Area X and retrogradely labelled cells in lMAN, respectively. Triangles indicate the implantation track (white), and the border of Area X (blue) and lMAN (red). D, dorsal; R, rostral. Scale bar, 250 µm.

(d) Spectrograms of song recorded from one adult bird implanted with microdialysis probes in Area X at baseline (infused with PBS) and on the third day of chronic infusion of DA, SKF or Qui. Black lines indicate motifs. Scale bar, 200 ms.

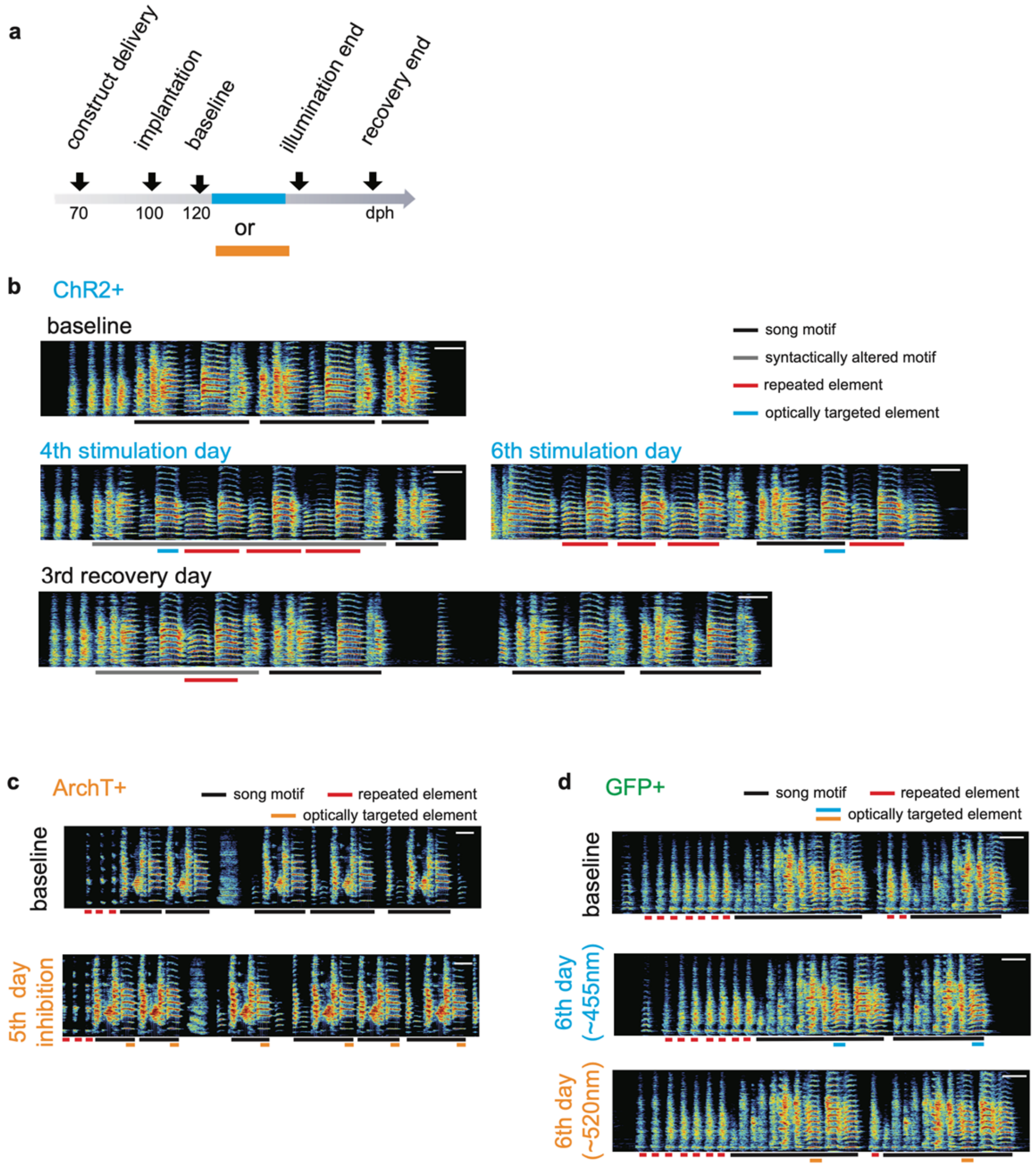

Supplementary Figure 12. Song contingent optical manipulation of dopamine terminals in Area X.
(a) Schematic of experimental design for optogenetic manipulation of dopamine release from VTA terminals. Optogenetic constructs were delivered into VTA in juvenile birds (~70 dph) and cannulas targeting Area X were not bilaterally implanted until the song crystallized (~100 dph). Optogenetic manipulations began at least 1-week post cannula implantation to allow birds to fully recover and to begin singing again (blue box, wavelength of LED ~455 nm; green box, wavelength of LED ~520 nm). Songs recorded before the illumination started and after the illumination ceased are referred to as baseline and recovery, respectively.
(b) Spectrograms of song recorded from a ChR2+ bird at baseline, on the 4th/6th stimulation day and on the 3rd recovery day. They show a bird repeating a pair of syllables within the song motif or in the beginning or end of the song motif. Scale bar, 200 ms.
(c-d) Lack of changes in vocal repetitions in birds expressing ArchT or GFP following optical manipulations. (c) Spectrograms of song recorded from a ArchT+ bird at baseline and on the 5th inhibition day. Light pulses (~520 nm, 100ms) were delivered over the target syllable in a subset of variants during inhibition days. Scale bar, 200ms. (d) Spectrograms of song recorded from a GFP+ bird at baseline and on the 6th day of optical illumination. Light pulses (middle, blue light, ~455nm, 100ms; bottom, green light, ~520 nm, 100ms) were delivered over the target syllable (blue or orange line) in a subset of variants during illumination days. Scale bar, 200ms.

REFERENCES

1. Saunders, A.*, et al.* Molecular Diversity and Specializations among the Cells of the Adult Mouse Brain. *Cell* **174**, 1015-1030 e1016 (2018).
2. Wallace, M.L.*, et al.* Genetically Distinct Parallel Pathways in the Entopeduncular Nucleus for Limbic and Sensorimotor Output of the Basal Ganglia. *Neuron* **94**, 138-152 e135 (2017).
3. Abdi, A.*, et al.* Prototypic and arkypallidal neurons in the dopamine-intact external globus pallidus. *J Neurosci* **35**, 6667-6688 (2015).
4. Papathanou, M., Dumas, S., Pettersson, H., Olson, L. & Wallen-Mackenzie, A. Off-Target Effects in Transgenic Mice: Characterization of Dopamine Transporter (DAT)-Cre Transgenic Mouse Lines Exposes Multiple Non-Dopaminergic Neuronal Clusters Available for Selective Targeting within Limbic Neurocircuitry. *eNeuro* **6**(2019).
