# Supplementary material for: Expression of FoxP2 in the basal ganglia regulates vocal motor sequences in the adult songbird
Source: Nat Commun. 2021 May 11;12:2617. doi: 10.1038/s41467-021-22918-2 (PMC8113549; doi:10.1038/s41467-021-22918-2)
Supplement: Supplementary file 3 — Reporting Summary [file 41467_2021_22918_MOESM3_ESM.pdf]

nature research

Corresponding author(s): Todd Roberts

Last updated by author(s): 3/22/2021

Reporting Summary

Nature Research wishes to improve the reproducibility of the work that we publish. This form provides structure for consistency and transparency in reporting. For further information on Nature Research policies, see our Editorial Policies and the Editorial Policy Checklist.

Statistics

For all statistical analyses, confirm that the following items are present in the figure legend, table legend, main text, or Methods section.

n/a	Confirmed	
☐	☒	The exact sample size (n) for each experimental group/condition, given as a discrete number and unit of measurement
☐	☒	A statement on whether measurements were taken from distinct samples or whether the same sample was measured repeatedly
☐	☒	The statistical test(s) used AND whether they are one- or two-sided<br>*Only common tests should be described solely by name; describe more complex techniques in the Methods section.*
☐	☒	A description of all covariates tested
☐	☒	A description of any assumptions or corrections, such as tests of normality and adjustment for multiple comparisons
☐	☒	A full description of the statistical parameters including central tendency (e.g. means) or other basic estimates (e.g. regression coefficient) AND variation (e.g. standard deviation) or associated estimates of uncertainty (e.g. confidence intervals)
☐	☒	For null hypothesis testing, the test statistic (e.g. F, t, r) with confidence intervals, effect sizes, degrees of freedom and P value noted<br>*Give P values as exact values whenever suitable.*
☒	☐	For Bayesian analysis, information on the choice of priors and Markov chain Monte Carlo settings
☒	☐	For hierarchical and complex designs, identification of the appropriate level for tests and full reporting of outcomes
☒	☐	Estimates of effect sizes (e.g. Cohen's d, Pearson's r), indicating how they were calculated

Our web collection on statistics for biologists contains articles on many of the points above.

Software and code

Policy information about availability of computer code

Data collection	Acoustic signals were recorded using Sound Analysis Pro2011(Tchernichovski, Nottebohm et al. 2000)<br>Fixed tissue images were collected using Zen 2 black edition (Zeiss, Germany) and and analyzed in ImageJ(1.52p).
Data analysis	Behavioral data were analyzed using Sound Analysis Pro2011(Tchernichovski, Nottebohm et al. 2000) and custom scripts in Matlab R2015A, with additional statistical analysis in Prism 6.0 (GraphPad Software, USA). Transcriptomic analayses were conducted in R (version 3.6.0).

For manuscripts utilizing custom algorithms or software that are central to the research but not yet described in published literature, software must be made available to editors and reviewers. We strongly encourage code deposition in a community repository (e.g. GitHub). See the Nature Research guidelines for submitting code & software for further information.

Data

Policy information about availability of data

All manuscripts must include a data availability statement. This statement should provide the following information, where applicable:

- Accession codes, unique identifiers, or web links for publicly available datasets
- A list of figures that have associated raw data
- A description of any restrictions on data availability

The NCBI Gene Expression Omnibus (GEO) accession number for the raw snRNA-sequencing data in this manuscript (figures 4-6) is GSE136086. Processed data for snRNA-sequencing analyses are available at https://cloud.biohpc.swmed.edu/index.php/s/nLicEtkmjGGmRF8. Code for snRNA-sequencing data, including pre-processing, clustering, differential gene expression analyses, and producing all related figures are available on GitHub (https://github.com/konopkalab/songbird_areax).. Source data for figures 1-3 and 7-8 are provided with the paper, all other data associated with this paper are available from the corresponding author upon reasonable request.

Field-specific reporting

Please select the one below that is the best fit for your research. If you are not sure, read the appropriate sections before making your selection.

☒ Life sciences ☐ Behavioural & social sciences ☐ Ecological, evolutionary & environmental sciences

For a reference copy of the document with all sections, see nature.com/documents/nr-reporting-summary-flat.pdf

Life sciences study design

All studies must disclose on these points even when the disclosure is negative.

Sample size	No a priori sample size calculation was performed. We chose sample sizes that are comparable to similar experiments conducted by others in the field. For behavioral experiments, at least 4 animals were used for each condition for at least 5 data-points in each comparison to enable statistical analysis. For the snRNA-seq experiments, two animals were used for each condition and tissue samples are pooled for the data analysis. Sample sizes for snRNA-seq statistics are robust because the statistical units are cells (total n > 27,000), not individual birds. For other experiments, sample size n>=3.
Data exclusions	Data were excluded in experiments where viral injections were performed and post-hoc analysis of viral targeting demonstrated that injections were inaccurate.
Replication	Replication was extensive throughout the manuscript. All main results were repeated in multiple animals with exact n numbers for each experiment provided in the manuscript. We define each animal as comprising an independent experiment. No issues were identified in replicating any of the reported findings.
Randomization	Birds subjected to optogenetic manipulation were pre-screened because the voice detection algorithm we use cannot do on-line detection of all syllables or syllables from all birds. As described in our previous work (Xiao et al, 2018), the false positive/negative targeting rates need to be maintained under 10%. The birds with songs that do not meet this threshold were not used. Otherwise, birds set up for other experiments were randomly assigned.
Blinding	For snRNA-seq experiments, blinding was not relevant to this study as all experimental data was included for analysis and procedures were standardized. For behavioral experiments, investigators were not blinded to allocation during data collection because data was collected in an automated and unbiased way, thus there was no subjective manipulation possible by the experimenter. Investigators were blinded to allocation and outcome assessments during analysis in all experiments.

Reporting for specific materials, systems and methods

We require information from authors about some types of materials, experimental systems and methods used in many studies. Here, indicate whether each material, system or method listed is relevant to your study. If you are not sure if a list item applies to your research, read the appropriate section before selecting a response.

Materials & experimental systems

n/a	Involved in the study	
☐	☒	Antibodies
☐	☒	Eukaryotic cell lines
☒	☐	Palaeontology and archaeology
☐	☒	Animals and other organisms
☒	☐	Human research participants
☒	☐	Clinical data
☒	☐	Dual use research of concern

Methods

n/a	Involved in the study	
☒	☐	ChIP-seq
☒	☐	Flow cytometry
☒	☐	MRI-based neuroimaging

Antibodies

Antibodies used	The primary antibodies used were: Goat anti-FOXP2 (1:500, ab1307, Abcam, MA, USA), Goat anti-FOXP2 (1:500, sc-21069, Santa Cruz Bio., TX, USA), mouse anti-V5 tag (1:2000, R960-25, Invitrogen, CA, USA), rabbit anti-RFP (1:1000, mCherry, 600-401-379, Rockland, PA, USA), mouse anti-RFP (1:1000, mCherry, 200-301-379, Rockland, PA, USA), rabbit anti-GFP (1:2000, A11122, Invitrogen, CA, USA), chicken anti-GFP (1:1000, AB16901, Millipore, MA, USA), rabbit anti-tRFP (1:200, tagBFP, AB233, Evrogen, Moscow, Russia), mouse anti-GAPDH (1:20000, MAB374, Millipore, MA, USA) and rabbit anti-beta Tubulin(1:20000, ab6046, Abcam MA, USA).<br>The secondary antibodies used were: anti-chicken Alexa Fluor 488,703-545-155, 1:500; anti-rabbit DyLight 405,711-475-152, 1:500; anti-goat Alexa Fluor 647,705-605-003, 1:500; anti-mouse Alexa Fluor 594,711-585-150, 1:500 or anti-rabbit Alexa Fluor 594,711-585-152, 1:500(Jackson Immuno Research, ME, USA). Horseradish peroxidase (HRP)-conjugated species-specific secondary antibodies (1:10000, anti-rabbit NA934 and anti-mouse NA931, Sigma-Aldrich, MO, USA; 1:10000, anti-goat AP180P, Millipore, MA, USA)
Validation	All antibody validations are available on manufacturers' websites or previous published studies, Xiao et al., 2018.The specificity of

primary antibodies against FoxP2, mCherry or GFP were confirmed by two independent primary antibodies for both Immunohistochemistry (in zebra finch) and Immunoblotting (in cell cultures). tRFP antibody was validated for both Immunohistochemistry (in zebra finch) and Immunoblotting (in cell cultures). V5, GAPDAH and Tubulin antibodies were validated for Immunoblotting in cell cultures.

Eukaryotic cell lines

Policy information about cell lines

Cell line source(s)	HEK293T(ATCC)
Authentication	Cell lines used in this study was not authenticated.
Mycoplasma contamination	Cell lines used in this study was not tested for mycoplasma contamination.
Commonly misidentified lines (See ICLAC register)	HEK293T stable cell lines expressing vehicle or Cre-GFP were generated to validate CS constructs in vitro.

Animals and other organisms

Policy information about studies involving animals; ARRIVE guidelines recommended for reporting animal research

Laboratory animals	Male zebra finch were used when they are older than 60 days post hatch; New born mice(C57Bl/6J, P0, unknown sex) were used for primary culture.
Wild animals	This study did not involve wild animals.
Field-collected samples	This study did not involve samples collected from the field.
Ethics oversight	All procedures were performed in accordance with established protocols approved by the UT Southwestern Medical Center Animal Care and Use Committee.

Note that full information on the approval of the study protocol must also be provided in the manuscript.
